# Radial Artery Calcification in Predicting Coronary Calcification and Atherosclerosis Burden

**DOI:** 10.1155/2022/5108389

**Published:** 2022-05-31

**Authors:** Alexandru Achim, Kornél Kákonyi, Ferenc Nagy, Zoltán Jambrik, Albert Varga, Attila Nemes, Jeffrey Shi Kai Chan, Gabor G. Toth, Zoltán Ruzsa

**Affiliations:** ^1^Second Department of Internal Medicine, Division of Invasive Cardiology, University of Szeged, Szeged, Hungary; ^2^“Niculae Stancioiu” Heart Institute, University of Medicine and Pharmacy “Iuliu Hatieganu”, Cluj-Napoca, Romania; ^3^Heart Failure and Structural Heart Disease Unit, Cardiovascular Analytics Group, Hong Kong, China; ^4^University Heart Center Graz, Department of Cardiology, Medical University of Graz, Graz, Austria

## Abstract

**Background:**

Atherosclerosis is a systemic arterial disease with heterogeneous involvement in all vascular beds; however, studies examining the relationship between coronary and radial artery calcification are lacking. The purpose of this study was to assess the relationship between the two sites and the prognostic value of radial artery calcification (RC) for coronary artery disease.

**Methods:**

This is a single-center, retrospective cross-sectional study based on Doppler ultrasound of radial artery (RUS) and coronary artery angiography (CAG). We included a total of 202 patients undergoing RUS during distal radial access and CAG at the same procedure, between December 2020 and May 2021, from which 103 were found having RC during RUS (RC group) and 99 without (NRC group). Coronary calcifications were evaluated either by angiography examination (moderate and severe), positive CT (>100 Agatson units), or intracoronary imaging (IVUS, OCT).

**Results:**

A significant correlation was observed between radial calcification and coronary calcification variables (67.3% vs. 32.7%, *p*=0.001). The correlation between risk factors such as age, smoking, chronic kidney disease, and diabetes mellitus was higher while sex did not play a role. The need of PCI and/or CABG was higher in the RC group (60% vs. 44%, *p*=0.02). RC, therefore, predicts the extent and severity of coronary artery disease.

**Conclusion:**

RC may be frequently associated with calcific coronary plaques. These findings highlight the potential beneficial examination of radial arteries whenever CAD is suspected.

## 1. Introduction

Asymptomatic individuals with significant coronary artery disease (CAD) are at risk of unanticipated cardiac events including myocardial infarction (MI). Laboratory studies, stress tests, and coronary artery imaging including coronary artery calcification (CAC) scoring are used for evaluating at-risk individuals. CAC scoring has been demonstrated to not only show current coronary disease but also predict future cardiac events [1–3]. Coronary artery calcification and cardiac valve calcific deposits correlate well and predict mortality in the general population [4, 5]. There also seems to be a strong association between carotid and coronary stenosis [6–9]. While carotid examination in CAD and vice versa has become of clinical importance in order to accurately identify patients who could benefit from aggressive preventive therapies as well as timely treatment, no relationship between radial and coronary arteries has been investigated. Based on the shared underlying atherosclerosis pathology in the two arterial systems, this study aimed to explore whether the extent of calcifications in the two arteries is correlated and if RC is a parameter for predicting CAD.

## 2. Methods

### 2.1. Study Population and Design

**Figure 1:**
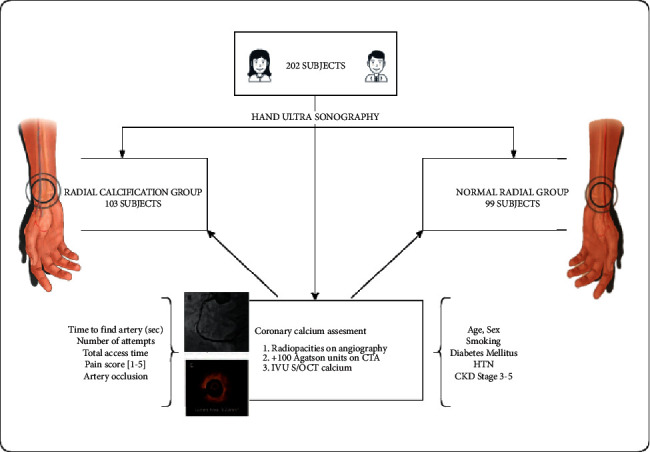
Study design and patient selection. Inclusion in each group was done blindly and retrospectively.

From December 2020 to May 2021, 202 consecutive patients who underwent coronary angiography and who required radial Doppler ultrasound examination were recruited in the study. All patients from this period who came to our catheterization laboratory for various transcatheter procedures were included, in the context in which they received standardized pre- and postoperative RUS evaluation of the radial artery [10–12]; the only inclusion criterion was therefore the invasive cardiovascular evaluation, where the ultrasound images were clear, conclusive, and could be noted retrospectively. Design of the study is presented in Figure 1. The 2 groups were divided, according to the sonographic result at the level of the radial artery. Coronary status was analyzed as a follow-up. A correlation was made between the two arterial systems, with emphasis on the most relevant risk factors and the coronary outcome.

### 2.2. Calcification Assessment

**Figure 2:**
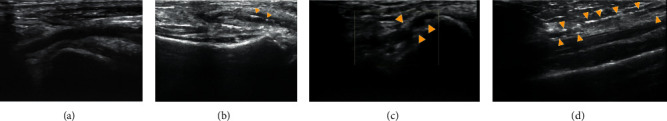
Ultrasound scanning of the distal radial artery, showing normal aspect (a) and calcific deposits within the vessel wall (yellow arrows), organized as calcific nodules (b), calcific plaques (c), and diffuse mediocalcinosis (d).

All patients underwent RUS-assisted distal radial puncture, as per center's protocol, scanning the artery at the anatomical snuffbox area, using a 7.5 MHz probe. Cross sections of the radial artery were assessed using the following factors: lumen diameter, vessel diameter, plaque distribution, and percent plaque area, with particular attention given to the type and extent of calcium deposition (diffuse vs. nodular, medial vs. intimal). RC was visually assessed accordingly, assigning scores in each of two calcification categories based on ultrasound findings, as follows: longitudinal involvement, 0 = no calcification, 1 = focal calcification, and 2 = diffuse calcification; density, 0 = no calcification, 1 = light calcification, and 2 = dense calcification. The designation of light versus dense calcification was purely qualitative. A calcification index was derived and patients with a score of minimum 2 pcts were considered positive and included in the RC group. Only clear echoreflective areas with acoustical shadowing associated with calcific plaques, as exemplified in Figure 2, were included.

As step two, quantitative analysis of the angiographic images was performed by a single individual blinded to the ultrasound results. Positive coronary calcification was defined as one of the following: (1) on angiography, radiopacities readily visible but mild degree and/or obvious, heavy calcification seen without cardiac motion, before contrast injection; (2) on cardiac CT, calcium score above 100 Agatson units; (3) during intracoronary imaging (IVUS, OCT), the presence of an arc of calcium >180°, length >5 mm, and calcium thickness >0.5 mm.

Significant CAD was defined by the need of PCI and/or CABG. Additional risk factors (age, sex, smoking, diabetes mellitus, primary hypertension, and renal failure) and radial access performance indexes (time to find artery [sec], number of attempts, access time [sec], pain score [1–5], and artery occlusion) were analyzed.

### 2.3. Statistical Analysis

Continuous variables were expressed as mean ± standard deviation. Statistical analyses were performed using IBM SPSS v26.0 (Chicago, IL, USA). Correlations between dichotomious variables were performed using the Pearson Chi Squared test, or Fisher's test. Median values between the two groups were compared using Mann–Whitney *U* test. A multivariable logistic regression analysis was performed to identify independent predictors of RC. All *p* values were two-sided, and *p* < 0.05 was considered statistically significant.

Written informed consent was obtained from all patients, and the Institution's Ethics Committee approved the study.

## 3. Results

Baseline characteristics are presented in Table 1. There was no difference in sex across the two groups, but the mean age of the RC group was significantly higher (69.24 ± 9.80 years vs. 63.35 ± 11.59 years, *p*=0.001). The full spectrum of patients was included but the main indication for coronary angiography remained to be stable angina (40%).

Representative duplex ultrasound images of normal and calcified radial arteries are shown in Figure 2. The normal artery (Figure 2(a)) is characterized by a thin, homogeneous wall and a smooth, luminal surface. Calcifications (Figure 2(b)–2(d)) appear as echoreflective areas within the vessel wall (not to be confused with tissue streaking seen in the soft tissues of both normal and calcific studies) and are associated with acoustical shadowing. The calcified vessel in Figures 2(b)–2(d) is narrower in caliber and exhibits an irregular luminal surface.

There was a statistically significant association between the presence of radial calcinosis and coronal calcification (*p*=0.001). The usage of PCI and/or CABG was significantly higher in the patients with radial calcinosis (*p*=0.02) (Table 2).

Several comorbidities were evaluated. An unadjusted analysis was performed to establish the risk factors involved in the presence of the radial calcinosis (Table 3). Out of a total of 19 smokers, 16 (84.21%) of them presented radial calcinosis (*p*=0.001). Patients with renal failure had a higher frequency of renal calcinosis (69.23%, 45/65) than the patients without renal failure (42.33%, 58/137), the difference being statistically significant (*p*=0.001). A statistically significant correlation was established between the presence of renal calcinosis and diabetes (55.97%, 89/159 vs. 32.55%, 14/43, *p*=0.001). No statistically significant correlations between either hypertension or artery occlusion and the presence of radial calcinosis were found.

Afterwards, a multivariable logistic regression analysis was performed (Table 4), demonstrating that age over 60 (*p*=0.001, OR 3.4, 95% CI), smoking (*p*=0.03, OR = 4.9, 95% CI), renal failure (*p*=0.01, OR = 2.3, 95% CI), and diabetes (*p*=0.03, OR = 2.3, 95% CI) were independently associated with radial calcinosis.

A series of parameters involved in the performance of the radial puncture were compared between the two groups. The mean value of the time to find artery was significantly higher in the patients who presented radial calcinosis (median time 3 minutes vs. 2 minutes, *p*=0.01). There were no statistically significant differences regarding the number of attempts, access time, or pain score (Table 5).

## 4. Discussion

The main findings of our study were (1) significant correlation between radial and coronary calcification in adults presenting with angina symptoms and associated risk factors and (2) the rate of revascularization treatment was higher in this population, suggesting the potential of radial artery calcification to become a new marker of prediction of severe coronary artery disease.

Based on our study, we suggest that incidental findings of upper extremity artery calcification on routine radiographs or Doppler ultrasound may warrant systemic evaluation for atherosclerosis in other areas of the body, especially screening for CAD. Increasing RC occurrence correlated with CAC, but more importantly with more advanced CAD (60% rate of PCI/CABG in the RC group vs. 44% in the NRC group). Latest European prevention guidelines state that CAC scoring may be considered to improve risk classification, and plaque detection by carotid ultrasound is an alternative when CAC scoring is unavailable or not feasible (level of recommendation IIb) [13]. Thus, the theory of including RUS as another alternative is attractive.

Risk factors seem to play a role for arterial calcification. Our study confirmed that radial calcinosis is more frequently found in population above 60 years, smokers, diabetics, hypertensives, and chronic kidney disease patients, with a strong emphasis on smoking (4.8 times higher risk).

Our findings are clinically important for several other reasons. First, RUS may serve as a pre- and peri-procedural adjuvant tool for the interventionist, facilitating a “per primam” selection of coronary calcium debulking technique, intuiting stent underexpansion, and preparing the interventionist to expect a more difficult sheath placement or even radial access failure, with a longer, more complex procedure. Not losing the radial access advantages in complex PCIs of severe calcific disease is of paramount importance [14]. Second, RUS may be useful to cardiovascular surgeons, since the radial artery is commonly used as a conduit for coronary artery bypass and the presence of calcifications may reduce suitability of this graft. Third, the strong relationship we found between RC and severity of coronary artery disease and stenosis not only serves to predict the presence of severe disease, but also aids in the identification of patients demonstrating established arterial disease who need intensive risk factors control and follow-up management.

**Figure 3:**
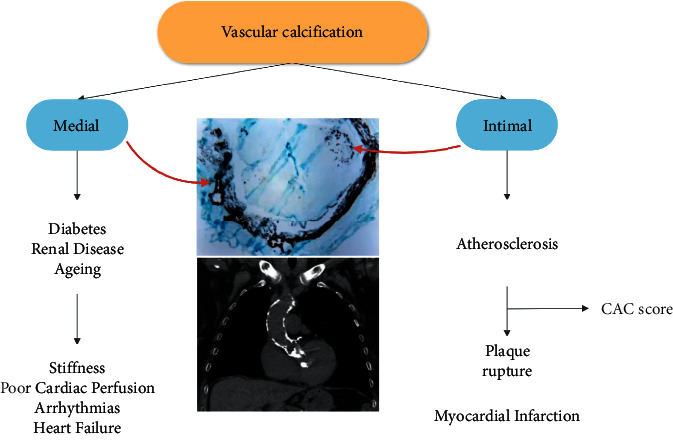
Site-specific phenotype of calcific lesions according to their location within the arterial wall.

For many decades, vascular calcification has been noted as a consequence of aging. Studies now confirm that vascular calcification is an actively regulated process and shares many features with bone development and metabolism. It occurs in two sites, the tunica intima and the tunica media, with different disease association and outcomes (Figure 3).

The intimal layer of the vessel wall is normally composed of endothelial cells and a small amount of subendothelial connective tissue. In atherosclerosis, the intima becomes greatly inflamed and thickened and calcification occurs. Natural history is that microcalcifications may arise inside lipid pool following the apoptosis of smooth muscle cells or macrophages. They coalesce into larger mases over time to form speckles, further progressing to calcified sheets or plates. Fragmentation of these sheets leads to nodules that may extend to the lumen and become protuberant with discontinuation of the endothelium [15]. Calcification of coronary arteries is an excellent predictor of atherosclerotic plaque burden and may contribute to atherosclerotic plaque rupture, though the connection between atherosclerotic plaque calcification plaque rupture is heavily debated. Several studies show a link between high CAC and risk of cardiac events and mortality, yet some studies have suggested that the most calcified plaques may be more stable, and that the plaques most vulnerable to rupture may be those which have a mixed composition of calcified and uncalcified tissue [16]. Indeed, unstable lesions are associated with focal calcium deposits that may be related to fibrous cap disruption [15]. Calcium in a spotty distribution has previously been observed, pathologically, in sudden coronary death victims [17]. While spotty calcification was more commonly associated with unstable plaques, extensive calcification was more common with stable plaques [17].

The medial layer of the vessel wall is composed of smooth muscle cells and elastin-rich extracellular matrix. Calcification of the media occurs preferentially along the elastic lamina, as opposed to the diffuse localization seen in intimal calcification, and is associated with diabetes, kidney disease, hypertension, and osteoporosis (also referred to as Monckeberg's sclerosis). The result of medial calcification is a stiffening of the artery wall, with an associated rise in blood pressure, and a higher risk of cardiovascular mortality than that of intimal artery calcification, because left ventricular strain, hypertrophy, and decreased myocardial perfusion during diastole appear as maladaptive mechanisms [16, 18, 19].

**Figure 4:**
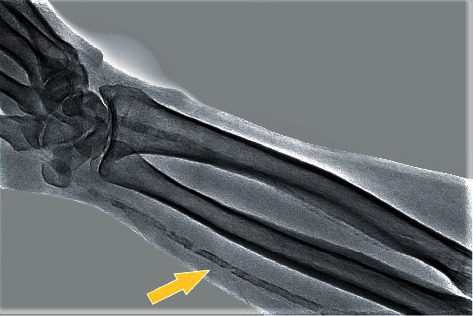
Forearm radiography: diffuse mediocalcinosis along the entire length of the radial artery (arrow).

At the same time, both layers can be affected simultaneously, with exponential harmful effect [20]. RUS can detect both forms of vascular calcification, as illustrated in Figure 2. Forearm fluoroscopy can also very obviously detect mediocalcinosis. An illustrative example is Figure 4, which shows how pregnant mediocalcinosis is and how distinctly it can be seen on a forearm X-ray. Such diffuse changes are most common in end-stage kidney disease. Our center is a dedicated ultrasound-assisted distal radial access center, having switched to this approach since 2019 [10–12]. Duplex US was used in the operating room to investigate all forearm arteries. RA diameter and peak systolic velocity were measured at the wrist level. We believe the use of ultrasound guidance enables the operator to identify important anatomical landmarks and avoid injuring adjacent structures. US can be also used to determine whether the lumen is large enough to accommodate the necessary sheath and check for calcifications that can block the equipment delivery. Therefore the RC aspect is also relevant for the operator's success as it can affect performance index. In our study, time of puncture and the number of attempts were similar across the two groups, but the total time to find the artery by US as well as the artery occlusion rate was higher in the radial calcification population (Table 5).

Vascular ultrasound-based imaging techniques allow relatively inexpensive and nonevasive widely available means to detect VC and to differentiate between Mönckeberg's medial calcific sclerosis and the atherosclerosis-related lesions and assess arterial wall abnormalities, such as intima-medial wall thickening and endothelial dysfunction [21]. This has been well described within peripheral arterial disease, predominantly chronic limb threatening ischemia [22] and carotid atherosclerosis [23] where US is a valuable tool for disease and risk assessment, indicated by the guidelines. Our findings are in line with the consistent evidence that VCs affects the entire arterial tree, adding another vessel to the puzzle and draws attention upon careful radial artery evaluation, especially when US is performed before and during cannulation anyhow.

### 4.1. Study Limitations

1. Although detecting subclinical atherosclerosis is valuable in risk stratification, we must acknowledge that direct proof that such detection translates into a better outcome is lacking, although several reports suggest that the frequency of use of risk-modifying interventions is increased when subclinical atherosclerosis is detected.
2. Although obtained in a small sample, these results indicate the usefulness of radial ultrasound as a further screening tool to identify patients who deserve consideration for a coronary noninvasive test. It is important to note, however, that this study was performed in patients who had undergone cardiac catheterization because of suspected or proved heart diseases; thus, whether our results can be extended to patients without a cardiovascular history remains to be defined.
3. Significant ischemic coronary disease was defined by the decision to continue with/history of PCI or/and CABG. Although coronary revascularization is a medical decision based on proven myocardial ischemia, not all significant coronary stenoses are followed by correct treatment and some nonsignificant coronary stenoses are overtreated.
4. It should be emphasized that the calcification scoring and evaluation is operator dependent, therefore subjective; however, we have adopted a policy of not exploring arteries that appear “borderline” or poor-quality images, projections, and so on; only clear calcific disease was counted as positive. Coronary angiography has low-moderate sensitivity compared to IVUS and CT for detection of CAC but is very specific (high positive predictive value) [24, 25].
5. Another limitation of the current study is the lack of histologic data to correlate with duplex findings. Histologic data would be helpful because the precise level of calcifications within the vascular wall cannot be determined by the imaging technique used, and the underlying pathology (atherosclerosis vs. Monckeberg's sclerosis), therefore, cannot be determined either.

## 5. Conclusion

RC may be associated with calcific coronary plaques frequently. These findings highlight the potential beneficial examination of radial arteries whenever CAD is suspected.

## Figures and Tables

**Table 1:** Baseline characteristics of all 202 patients.

Demographic features	Mean ± SD/N (%)		*p* value
	RC group (*n* = 103)	Non-RC group (*n* = 99)	
Age (years)	69.24 ± 9.80	63.35 ± 11.59	0.07
Gender: female/male, % (n)	43.6% (45)/56.3% (58)	40.4% (40)/59.6% (59)	0.44
Height (cm)	169.4 ± 8	169.05 ± 5	0.92
Weight (kg)	84 ± 15	87 ± 16	0.23
*Prior Comorbidities*			
Atrial fibrillation	17 (16.5%)	21 (21.2%)	0.39
Renal failure	45 (43.6%)	20 (20.2%)	0.003
Diabetes mellitus	89 (86.4%)	70 (70.7%)	0.006
Hypertension	45 (43.6%)	33 (33%)	0.13
Smoking	16 (15.5%)	3 (3.03%)	0.002
*Family History*	14 (13.6%)	11 (11.1%)	0.59
Dyslipidemia	29 (28.1%)	23 (23.2%)	0.42
Previous MI	12 (11.65%)	10 (10.1%)	0.72
Previous CABG	8 (7.7%)	3 (3.03%)	0.13
*Indication for Catheterization*			
Stable angina	42 (40.7%)	40 (38.8%)	0.95
Unstable angina	12 (11.6%)	8 (8.08%)	0.39
NSTEMI	22 (21.3%)	17 (17.1%)	0.45
STEMI	8 (7.7%)	14 (14.1%)	0.14
Heart failure	3 (2.9%)	2 (2.02%)	0.92
Severe aortic stenosis	5 (4.8%)	7 (7.07%)	0.45
Peripheral interventions	6 (5.8%)	8 (8.08%)	0.55
Other	9 (8.7%)	3 (3.03%)	0.32

CABG: coronary artery bypass graft; MI: myocardial infarction; NSTEMI: non-ST elevation myocardial infarction; RC: radial artery calcification; SD: standard deviation; and STEMI: ST elevation myocardial infarction.

**Table 2:** Association between the presence of coronary calcification and presence of radial calcinosis (top). Association between the usage of PCI and the presence of radial calcinosis (bottom).

Parameters		Radial calcinosis	No radial calcinosis	*p* value
Coronary calcification	Present	68	33	0.001
	Absent	35	66	
PCI/CABG	Used	62	44	0.02
	Not used	41	55	

CABG: coronary artery bypass graft; PCI: percutaneous coronary intervention.

**Table 3:** Unadjusted analysis of the risk factors involved in the presence of radial calcinosis.

Parameters		Radial calcinosis	No radial calcinosis	*p* value
Smoking	Smoker	16	3	0.001
	Non-smoker	87	96	
Renal failure	Absent	58	79	0.001
	Present	45	20	
Diabetes	Absent	14	29	0.001
	Present	89	70	
Hypertension	Absent	58	66	0.08
	Present	45	33	

**Table 4:** Multivariable logistic regression analysis of the risk factors involved in the presence of radial calcinosis.

	B	S.E.	Wald	df	Sig.	Odds ratio	Confidence interval
Age over 60	1.236	0.371	11.091	1	0.001	3.443	3.102–3.774
Smoking	1.453	0.669	4.711	1	0.03	4.875	3.921–6.118
Renal failure	0.855	0.346	6.095	1	0.014	2.352	2.091–2.797
Diabetes	0.845	0.402	4.424	1	0.035	2.328	1.762–3.111
Hypertension	0.579	0.311	3.294	1	0.07	1.764	1.394–2.122

B = beta coefficient; S.E. = standard error; Wald = the Wald test; df = degrees of freedom; and sig = statistical significance.

**Table 5:** Median values of the parameters involved in the performance of the radial punction (interquartile ranges).

Parameters	Radial calcinosis	No radial calcinosis	*p* value
Time to find artery (minutes)	3 (2, 10)	2 (1, 5)	0.01
Number of attempts	2 (1, 3)	2 (1, 2)	0.09
Access time (minutes)	37 (20, 60)	35 (20, 50)	0.16
Artery occlusion			
Absent	73	97	0.4
Present	4	2	

Between the two groups, artery lumen patency at 48 h follow-up, documented by RUS examination, showed a numerically higher occlusion rate in the RC group, which was not statistically significant (5.19% vs. 2.02%, *p*=0.4).

## Data Availability

The patient data used to support the findings of this study are available from the corresponding author upon request.

## Abbreviations

RC: Radial calcification
RUS: Radial ultrasound
CAG: Coronary artery angiography
CAD: Coronary artery disease
CT: Computer tomography
MI: Myocardial infarction
CAC: Coronary artery calcification
PCI: Percutaneous coronary intervention
CABG: Coronary artery bypass grafting
IVUS: Intracoronary vascular ultrasound
OCT: Optical coherence tomography.
